# Supplementary material for: Predicting Immunotherapy Outcomes in Older Patients with Solid Tumors Using the LIPI Score
Source: Cancers (Basel). 2022 Oct 17;14(20):5078. doi: 10.3390/cancers14205078 (PMC9600023; doi:10.3390/cancers14205078)
Supplement: Supplementary file 1 [file cancers-14-05078-s001.zip › cancers-1849807-supplementary.pdf]

LIPI and corticosteroid use

As shown in the multivariable Cox models, corticosteroid use and LIPI were independent prognostic factors for OS. We further investigated the prognostic value of LIPI in patients with or without corticosteroid use and found no difference in term of prognostication (Figure S1).

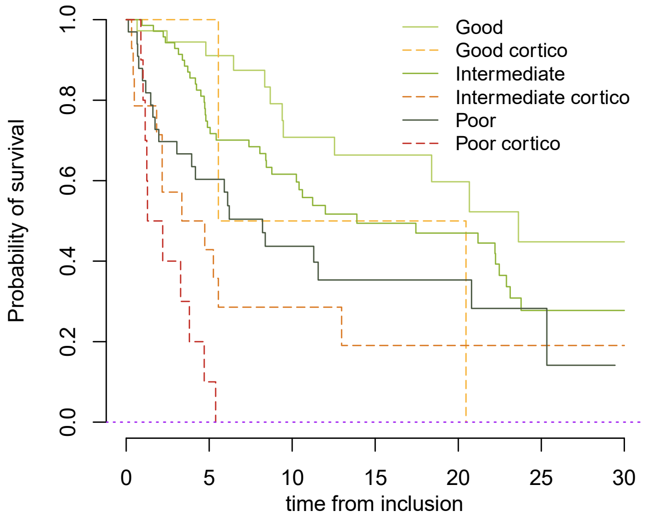

Figure S1. Response rates according to LIPI groups with or without corticosteroids.
